# Autism Spectrum Disorder Versus Autism Spectrum Disorders: Terminology, Concepts, and Clinical Practice

**DOI:** 10.3389/fpsyt.2020.00484

**Published:** 2020-05-25

**Authors:** Lindsay M. Oberman, Walter E. Kaufmann

**Affiliations:** ^1^Center for Neuroscience and Regenerative Medicine, Henry M. Jackson Foundation for the Advancement of Military Medicine, Rockville, MD, United States; ^2^Department of Human Genetics, Emory University School of Medicine, Atlanta, GA, United States

**Keywords:** autism (ASD), intellectual & developmental disabilities, genetics, syndrome, diagnosis, terminology

## Introduction

Autism spectrum disorder (ASD) is a behaviorally defined complex neurodevelopmental disorder. The diagnosis of ASD is based on observations and assessments of behavior using Diagnostic and Statistical Manual of Mental Disorders, Fifth Edition (DSM-5) (1) or International Classification of Diseases, 11th Edition (ICD-11) criteria (2). Though the DSM and ICD are quite useful in determining whether a given individual's behavior is consistent with a given diagnosis, it does not speak to the etiology or impact of co-occurring conditions on the behavioral phenotype or presentation. Genetic syndromes, defined mutations, and *de novo* copy number variations are reported to account for almost 10% to 20% of cases within ASD (3). While the revisions to the diagnostic criteria introduced a few years ago into DSM-5 (1) updated ASD from the conceptual and practical perspectives, some persistent confusion regarding terminology and the diagnosis of the condition in individuals with intellectual disability remains. The simplified diagnosis of ASD, which merged previous diagnoses into a single disorder, has led to its use in plural (autism spectrum disorders) for different purposes.

### From DSM-IV to DSM-5: Diagnosis of Autism Spectrum Disorder

The Diagnostic and Statistical Manual of Mental Disorders, Fourth Edition (DSM-IV) was released in 1994, at a time in which new knowledge on ASD was rapidly emerging. DSM-IV tried to systematize the different clinical entities associated with autistic features, including recently identified disorders, such as Rett syndrome (4). The category under which disorders with severe autistic features were grouped, pervasive developmental disorders included three disorders (i.e., autistic disorder, Asperger's disorder, PDD-NOS) with substantial clinical overlap. The category also included childhood disintegrative disorder and Rett syndrome, the latter a genetic disorder with initial descriptions of prominent autistic features (4). Despite text corrections on the PDD-NOS section in the subsequent DSM-IV revision (Diagnostic and Statistical Manual of Mental Disorders, Fourth Edition—Text Revision, DSM-IV-TR) (5), several major shortcomings were identified in the implementation of DSM-IV/DSM-IV-TR criteria (6). These included the consistency of the diagnosis of Asperger's disorder, frequently labeled as high-functioning autistic disorder; the adequacy of the use of the diagnosis of PDD-NOS for mild neurodevelopmental disorder and Asperger's disorder for individuals with unusual behaviors but not severe autistic features. Validity of the pervasive developmental disorders category and the diagnosis of childhood disintegrative disorder were also raised over the years (1), as well as new knowledge on the phenotype of Rett syndrome differentiated this entity from autistic disorder (7). The fact that the Centers for Disease Control and Prevention (CDC) reported rates of ASD by grouping by pervasive developmental disorders category (8), rather than by individual diagnoses, also contributed to the revisions of the DSM-IV approach to diagnosis. DSM-5 introduced three major changes: 1. It merged all of the diagnoses that were under the pervasive developmental disorders category into a single disorder termed autism spectrum disorder. 2. It eliminated two diagnostic entities (childhood disintegrative disorder and Rett syndrome). 3. It merged DSM-IV's Social and Communication symptom domains into a single social communication (and interaction) domain. Thus, DSM-5 recognized the empirical evidence demonstrating the challenges of implementing previous diagnostic schemes and the increasing body of literature supporting ASD as a broad spectrum diagnostic entity (1, 6).

### Autism Spectrum Disorder Versus Autism Spectrum Disorders

Prior to the introduction of the unitary diagnosis of autism spectrum disorder (ASD), the term autism spectrum disorders began to be applied to epidemiological studies (8) and, more commonly, in the basic science and genetics literature to refer to genetic disorders associated with prominent autistic features or with a relatively high proportion of individuals meeting ASD diagnostic criteria. Although the rationale for this disorder grouping is strong for research on molecular and neurobiological mechanisms in ASD, the specificity of the label and its clinical application are troublesome. While some autism spectrum disorders are characterized by a higher prevalence of ASD than the general population, this is usually not higher than 50% or severe autistic behaviors are only transient (e.g., Rett syndrome) (9). Moreover, there is broad overlap between the cellular processes underlying ASD and those responsible for intellectual disability and severe language impairment (10, 11). The clinical use of the term autism spectrum disorders is included in the diagnostic evaluation of certain genetic disorders with prominent autistic features in order to ensure that the child receives appropriate support services, including early intervention and behavior management. Proper neuropsychological evaluations are also useful in determining whether pharmacologic and non-pharmacologic therapies are appropriate as well as in guiding appropriate school/educational placement. As mentioned above, although Rett syndrome was included in DSM-IV's pervasive developmental disorders category (5), the DSM-5 Neurodevelopmental Disorders Working Group determined that, despite transient severe autistic features in Rett syndrome, there was no reason for selecting Rett Syndrome over the other genetic disorders associated with ASD. Thus, rather than creating an extremely long and rapidly obsolete list of disorders, the DSM-5 Neurodevelopmental Disorders Working Group decided it was better to consider the etiology of ASD (genetic or not) as a specifier (*Associated with a known medical or genetic condition or environmental factor*), which could further refine the diagnosis.

Another application of the term autism spectrum disorders is to emphasize that ASD is thought to be more than one disorder from the pathophysiological viewpoint. However, if ASD is considered a “broad” behavioral syndrome and, as DSM-5 stresses through its etiology specifier, an entity with multiple causes and mechanisms, we suggest that there is no need for a **plural** term. It does not add diagnostic value from the etiological viewpoint. If referring to heterogeneity in terms of cognitive or behavioral impairments, three key specifiers are also included in the DSM-5 ASD diagnosis: “With or without accompanying intellectual impairment,” “With or without accompanying language impairment,” and “Associated with another neurodevelopmental, mental, or behavioral disorder.” The single broad entity of ASD as defined by the DSM-5 is supported by field trials establishing the reliability, sensitivity, and specificity of the diagnosis (12). Meanwhile, the term Autism Spectrum Disorders is reminiscent of the five diagnoses in DSM-IV, diagnoses that ultimately demonstrated low consistency. Thus, although to some extent cumbersome, we suggest that the use of ASD plus the abovementioned specifiers is a better (i.e., more clear and specific) alternative to the term autism spectrum disorders.

### Autism Spectrum Disorder and Intellectual Disability

While the discussion about the term autism spectrum disorders underscores the strengths of DSM-5's definition of ASD and its associated recommendations, the guidelines appear to be inadequate for addressing social communication impairments associated with genetic disorders that often lead to various degrees of intellectual disability. In fact, it was recently noted that the difficulties in assigning an ASD diagnosis to an individual with a complex genetic syndrome were recognized many years ago by Leo Kanner (13). DSM-5 indicates that the diagnosis of ASD in intellectual disability is possible, as long as the autistic features cannot be explained by global intellectual or communication impairments: “social communication should be below that expected for general developmental level.” This statement puts emphasis not only on the selectivity of the deficits but also on the social communication and interaction impairment of ASD, which has led to some diagnostic challenges. ASD's core symptoms also include the presence of restricted and repetitive behaviors, interests, and activities (RRBs). Although all three types of social communication and interaction deficits (i.e., deficits in social-emotional reciprocity; deficits in nonverbal communicative behaviors used for social interaction; deficits in developing, maintaining, and understanding relationships) are required for the diagnosis, two of the four types of RRBs (i.e., stereotyped or repetitive motor movements, use of objects, or speech; insistence on sameness, inflexible adherence to routines, or ritualized patterns or verbal nonverbal behavior; highly restricted, fixated interests that are abnormal in intensity or focus; hyper- or hyporeactivity to sensory input or unusual interests in sensory aspects of the environment) are essential in achieving the high sensitivity and specificity of DSM-5's ASD criteria. Consequently, the best way to test the feasibility and adequacy of ASD criteria in intellectual disability is to apply them to well-defined groups. This is necessary because of the heterogeneity of cognitive impairment in the general population.

Evidence is emerging that the behavioral profile of ASD phenomenology is atypical in individuals with co-occurring genetic disorders (14–16). Two of the most common intellectual disability syndromes associated with ASD have been evaluated in terms of DSM-5 criteria, and they have revealed opposite diagnostic challenges. Wheeler and colleagues (17) reported that 86.4% of males and 61.7% of females with fragile X syndrome met DSM-5 criteria for RRBs but only 29.4% of males and 13.0% of females met criteria for the social communication and interaction domain, in contrast with previous reports of up to 60% males being diagnosed with ASD (18–20). By using the Autism Diagnostic Interview-Revised (ADI-R) along with DSM-5 criteria, we demonstrated that in Phelan-McDermid syndrome 90% individuals met the social communication and interaction criteria and 55% met the RRBs criteria (15). Nevertheless, the cohort did not demonstrate greater impairment in adaptive social skills than in adaptive communication skills in the Vineland Adaptive Behavior Scales, Second Edition (21), which raises questions about the validity of the ASD diagnosis in this disorder as it is not clear that the social communication deficits are below that expected for general developmental level. The discrepancies between these studies and previously reported prevalence figures can be analyzed in different ways. By comparing these DSM-5 analyses with the prevalence of ASD according to DSM-IV, an interpretation is that the lower figure in fragile X syndrome reflects DSM-5's lower sensitivity. However, this assumes that the accuracy of DSM-IV is greater or DSM-IV–based figures are the gold standard. Another interpretation is that other features of these genetic conditions lead to an over- or under-recognition of DSM-5 criteria. In support of the latter are studies reporting the complexity of behavioral and other associated impairments in individuals with fragile X syndrome and ASD diagnosis, in particular, the frequent anxiety co-morbidity (20, 22). **Table 1** lists cognitive and behavioral features that potentially lead to the overdiagnosis of ASD in fragile X syndrome and other genetic syndromes associated with intellectual disability (23).

**Table 1:** Cognitive and behavioral features affecting social communication in intellectual disability.

Communication
Concrete or inflexible thinking Difficulties with flow of conversation Difficulties with logical thinking Language disorder (e.g., reduced vocabulary) Stereotyped or repetitive speech* Reduced facial expression and gestures due to motor impairment (e.g., hypotonia, parkinsonism) Decreased pointing due to motor impairment (e.g., hypotonia or hypertonia, reduced hand function, poor motor coordination) Reduced eye contact (e.g., eye gaze avoidance)**
Behavior
Anxiety, general, or social types Hyperactivity or impulsiveness Sensory over-reactivity Irritability Repetitive movements (e.g., body rocking, hand flapping)* Perseverative behavior*

*Common behaviors in intellectual disability, which could be diagnostic RRBs for ASD and/or stereotypic movement disorder.

**Common behavior in fragile X syndrome with or without ASD; related to anxiety.

Given that the core social and behavioral symptoms of ASD may present differently in an individual with co-occurring intellectual disability and/or genetic disorders, the applicability and validity of standard ASD screening and diagnostic assessments and their standard scoring systems in these populations should be considered. Derks and colleagues were able to identify a specific subset of questions on the Social Communication Questionnaire (SCQ) that discriminated individuals with ASD and intellectual disability from those with intellectual disability alone (24). Additionally, our group recently adapted two commonly used ASD screening instruments, the Social Communication Questionnaire (SCQ) and the Social Responsiveness Scale-2 (SRS-2), for the diagnosis of ASD in fragile X syndrome. Our findings illustrate the difficulties in differentiating autistic features from characteristic cognitive and behavioral impairments observed in individuals with fragile X syndrome and other forms of intellectual disability (23). The study demonstrated that many SCQ and SRS-2 items are not sensitive to DSM-5 ASD diagnostic status. Furthermore, eliminating these non-specific items only leads to a modest increase in accuracy of the diagnosis of ASD in individuals with fragile X syndrome (23). Thus, it seems that in the context of genetic syndromes, the overall diagnostic impression of ASD is confounded by language impairment and other abnormal behaviors characteristic of neurodevelopmental genetic syndromes. The use of behavioral instruments, such as the Aberrant Behavior Checklist in Down syndrome with and without ASD (25), has also supported this notion. At this point, it is unclear whether these studies in genetic syndromes are applicable to non-syndromic ASD with intellectual disability. Evaluations of DSM-5 versus DSM-IV ASD criteria have demonstrated a lower overall prevalence of ASD using DSM-5 criteria, but greater agreement between DSM-IV and DSM-5 among individuals with intellectual disability than in those with normal cognition (26).

Unquestionably, many individuals with intellectual disability have social communication and interaction impairments that require adequate diagnosis and treatment. Nonetheless, the exclusive use of the ASD label in this situation decreases the validity of the ASD diagnosis with negative implications for clinical practice and research. If RRB-like features are not present at DSM-5's diagnostic threshold, we propose to use the more appropriate diagnosis of Social (Pragmatic) Communication Disorder (1). Although this entity was delineated as a specific communication disorder affecting the social communication domain, it includes most of the features and functional implications of the social communication and interaction deficits in ASD. In the situation where social communication deficits do not meet DSM-5 threshold, but the individual displays RRB-like features, we recommend using the label Stereotypic Movement Disorder (9). This entity was delineated with a focus on the frequently present repetitive movements and behaviors in intellectual disability, including genetic syndromes, such as fragile X syndrome. We hope future revisions of DSM will address this apparent over-diagnosis of ASD in intellectual disability and determine if the problem extends beyond genetic syndromes.

## Discussion

We conclude that the use of the term Autism Spectrum Disorders, to refer to genetic disorders associated with prominent autistic features, is not recommended. Use of the plural term. Use of the term Autism Spectrum Disorders is also problematic in clinical practice since DSM-5's framework takes into consideration the range of impairments and severity in ASD. We also suggest that the diagnosis of ASD is inaccurate in many individuals with intellectual disability, particularly in those with genetic syndromes where the social communication deficits are not below that which can be expected given the individual's developmental level. Rather than basing the diagnosis exclusively on the social communication and interaction impairments, we recommend employing other diagnostic entities in DSM-5, such as Social Communication Disorder or Stereotypic Movement Disorder as appropriate.

## Author Contributions

LO participated in the interpretation and drafting of results and drafted the manuscript. WK conceived of the study, participated in the interpretation and drafting of results, and drafted the manuscript.

## Funding

Work by the authors on Phelan-McDermid syndrome and fragile X syndrome and was supported, respectively, by a Seed Grant from the Simons Center for the Social Brain, at the Massachusetts Institute of Technology, and by cooperative agreements U01DD000231, U19DD000753, and U01DD001189, funded by the Centers for Disease Control and Prevention. This article's contents are solely the responsibility of the authors and do not necessarily represent the official views of the Centers for Disease Control and Prevention or the Department of Health and Human Services.

## Conflict of Interest

The authors declare that the research was conducted in the absence of any commercial or financial relationships that could be construed as a potential conflict of interest.
